# Repeated voriconazole therapeutic drug monitoring in hospitalised patients: a real-world cohort study of out-of-range exposure and concentration response to dose adjustment

**DOI:** 10.3389/fphar.2026.1867435

**Published:** 2026-09-02

**Authors:** Tian He, Zhang Liu, Limian Liang, Wenmei Qiao, Shiyun Chen, Jiajia Liu, Miaona Liu, Wei Li

**Affiliations:** 1 Department of Pharmacy, Shenzhen Third People's Hospital, Second Affiliated Hospital of Southern University of Science and Technology, National Clinical Research Center for Infectious Diseases, Shenzhen, Guangdong, China; 2 College of Pharmacy, Shenzhen Technology University, Shenzhen, China

**Keywords:** *CYP2C19*, dose adjustment, hospitalised patients, inflammation, therapeutic drug monitoring, voriconazole

## Abstract

**Background:**

Longitudinal evidence on repeated voriconazole therapeutic drug monitoring (TDM) in selected hospitalised patients is limited. We examined factors associated with out-of-range exposure and described concentration changes after dose adjustment in hospitalised patients undergoing repeated TDM.

**Methods:**

This retrospective, single-centre, longitudinal cohort study included hospitalised adults who received voriconazole and contributed at least three valid TDM episodes. Trough concentrations were classified as subtherapeutic (<1.0 μg/mL), in range (1.0–5.0 μg/mL), or supratherapeutic (>5.0 μg/mL). Bayesian mixed-effects logistic regression with weakly informative priors and patient-level random intercepts identified factors associated with each direction of out-of-range exposure. Adjacent TDM pairs with a documented dose change were analysed for return to range.

**Results:**

A total of 140 patients contributed 497 episodes; 72 (14.5%) were subtherapeutic and 145 (29.2%) supratherapeutic. In the 445-episode complete-case models, subtherapeutic exposure was associated with rifampin-like inducer co-administration (OR 16.48, 95% CrI 2.38–139.44) and lower daily dose (OR 0.45 per 100 mg, 95% CrI 0.29–0.66), whereas supratherapeutic exposure was associated with the *CYP2C19* poor-metaboliser phenotype (OR 5.24, 95% CrI 1.76–16.66), higher log-CRP (OR 1.69, 95% CrI 1.23–2.40), and higher total bilirubin (OR 1.09 per 10 μmol/L, 95% CrI 1.03–1.15). Concentrations returned to range after 7 of 12 (58.3%) clinically concordant dose increases, but after only 26 of 75 (34.7%) clinically concordant dose decreases.

**Conclusion:**

In hospitalised patients undergoing repeated voriconazole TDM, the factors associated with subtherapeutic and supratherapeutic exposure differed by direction. After dose reduction, supratherapeutic concentrations frequently did not return to range; this descriptive pattern is compatible with several contributors, including resampling before a new steady state and voriconazole’s nonlinear metabolism. These findings may help clinicians interpret repeated TDM results in complex inpatient settings.

## 1 Introduction

Voriconazole is widely used for invasive aspergillosis and other serious fungal infections (Herbrecht et al., 2002). Its pharmacokinetics are highly variable, shaped by *CYP2C19* genetic polymorphisms, hepatic function, inflammatory status, route of administration, and drug–drug interactions (Moriyama et al., 2017). These factors can change during treatment. Inflammation has attracted particular attention: proinflammatory cytokines suppress hepatic CYP activity and can shift a patient’s effective metabolic phenotype regardless of genotype, a process increasingly referred to as phenoconversion (Boglione-Kerrien et al., 2023). The baseline prevalence of *CYP2C19* poor-metaboliser phenotype varies by ancestry, occurring in approximately 15%–20% of Asian populations compared with 3%–5% of White populations (Dean, 2019). Because voriconazole has a narrow therapeutic window and undergoes nonlinear, saturable metabolism, even modest changes in clearance can move concentrations into ranges associated with treatment failure or toxicity (Dolton and McLachlan, 2014). TDM is therefore recommended by international guidelines for patients at risk of variable exposure (Patterson et al., 2016).

Most published voriconazole TDM studies are observational and rely on single trough concentration measurements (Li et al., 2024). Recent data from a Chinese cohort showed that inflammation can mask the effect of *CYP2C19* genotype on voriconazole metabolism (Hao et al., 2023), and population pharmacokinetic models incorporating CRP as a time-varying covariate have improved clearance predictions (van den Born et al., 2023). These advances, however, have not addressed repeated TDM in the longitudinal care of individual hospitalised patients. In that setting, two questions arise. Are the factors associated with subtherapeutic exposure the same as those associated with supratherapeutic exposure? And when a clinician adjusts the dose, how often does the next concentration actually return to the target range?

Most individual factor–exposure relationships in voriconazole TDM are already established, so the contribution here is descriptive rather than aimed at identifying new determinants. Prior longitudinal analyses of repeated voriconazole monitoring exist but have largely been confined to specific populations, such as allogeneic haematopoietic stem-cell transplant recipients (Gautier-Veyret et al., 2015); how repeated monitoring behaves across a general inpatient population, and whether dose adjustment reliably corrects exposure, are less well described. We therefore conducted a retrospective longitudinal cohort study of hospitalised patients who underwent repeated voriconazole TDM in routine care, focusing on factors associated with each direction of out-of-range exposure across multiple monitoring episodes and on concentration changes following dose adjustment.

## 2 Methods

### 2.1 Study design and setting

This single-centre, retrospective, longitudinal cohort study was conducted at the National Clinical Research Center for Infectious Diseases, The Third People’s Hospital of Shenzhen. The hospital is a tertiary referral centre with dedicated services for respiratory infections, organ transplantation, HIV/AIDS, and hepatic disease, and includes routine clinical pharmacy support for voriconazole TDM. The study period was from 1 January 2022 to 16 March 2026 (database lock date). Reporting followed the STROBE statement for observational studies (von Elm et al., 2008). Ethical approval was obtained from the institutional Research Ethics Committee before study initiation (Approval No. 2025–220-02). All patients who underwent institutional pharmacogenetic testing had signed informed consent at the time of clinical testing or biobank enrolment. For the retrospective analysis of de-identified clinical data from the remaining patients, the requirement for additional informed consent was waived.

### 2.2 Study population

Eligible patients were hospitalised adults who received voriconazole in routine care and contributed at least three valid TDM episodes during a treatment course. No patient under 18 years was included (youngest 23 years). This threshold was prespecified to ensure enough within-patient repeated measurements for mixed-effects modelling and to focus on the subgroup in whom repeated monitoring reflected persistent clinical uncertainty about exposure. Additional inclusion criteria were availability of basic demographic data and at least one episode with identifiable dosing information. Episodes were excluded if concentration results could not be confirmed, sampling times could not be assigned to a treatment course, or key dosing information could not be recovered after manual review. To limit the influence of a few very long monitoring series and to keep the analysis centred on the early-to-mid management phase of repeated TDM, we applied a prespecified maximum of six valid episodes per patient.

### 2.3 Voriconazole concentration measurement and episode definition

Voriconazole concentrations were measured by the hospital clinical laboratory using high-performance liquid chromatography (HPLC; 97.5% of measurements) or liquid chromatography–tandem mass spectrometry (LC–MS/MS; 2.5%), standardised to μg/mL. The lower limits of quantification were 0.48 μg/mL for HPLC and 0.05 μg/mL for LC–MS/MS; concentrations below the applicable lower limit were retained with a flag and classified as subtherapeutic in categorical analyses. A TDM episode was valid when the sample was clinically interpretable as a trough, the preceding regimen could be confirmed from structured medication records, and the assay result could be standardised. Trough sampling was defined pragmatically as blood drawn within approximately 1 hour before the next scheduled dose, or during early-morning phlebotomy in patients on stable twice-daily regimens. A precise-trough flag and a steady-state flag were recorded for each episode (Supplementary Methods). All included records were manually cross-verified by clinical pharmacists.

### 2.4 Outcomes

The primary outcomes were subtherapeutic exposure (<1.0 μg/mL) and supratherapeutic exposure (>5.0 μg/mL) at each valid TDM episode, defined according to a target trough range of 1–5 μg/mL. This range is consistent with British Society for Medical Mycology guidelines (Ashbee et al., 2014), which recommend trough concentrations >1 mg/L for efficacy and <4–6 mg/L to minimize toxicity. The conservative upper limit of 5.0 μg/mL was adopted to further reduce toxicity risk (Jin et al., 2016). The secondary outcome was return to the target range after a documented dose adjustment between two adjacent valid episodes. Clinically concordant pairs were prespecified as subtherapeutic episodes followed by dose increase and supratherapeutic episodes followed by dose decrease.

### 2.5 Covariates

Covariates were preselected on clinical-pharmacological grounds and grouped into genetic, hepatic, inflammatory, drug-interaction and treatment-related domains. *CYP2C19* genotyping used a fluorescence hybridisation-based assay (Ling et al., 2024) detecting the *2 (c.681G>A), *3 (c.636G>A) and *17 (c.-806C>T) alleles. Metaboliser phenotypes (NM, RM, IM, PM) were assigned per CPIC guidelines (Moriyama et al., 2017); the two patients carrying *1/*17 were classified as rapid metabolisers (RM). Genotype results were obtained retrospectively from clinical records and the institutional biobank; all patients who underwent institutional pharmacogenetic testing, including biobank enrolment, had signed informed consent at the time of testing/sample banking. For patients without an existing clinical genotype record, inclusion in the genotype-based analyses depended on whether genotype information could be ascertained through the institutional clinical or biobank framework permitted by the applicable ethics and consent procedures; patients for whom genotype information could not be ascertained were not included in the genotyped cohort. Of 151 patients meeting the monitoring criteria, 140 (92.7%) had genotype results and were included. Because alleles beyond *2, *3 and *17 were not assessed, rapid or ultrarapid misclassification cannot be fully excluded, although this is most relevant to fast-metaboliser classification and less so to the poor-metaboliser findings emphasised here.

To compare both directions of out-of-range exposure on the same basis, both models used the same covariate pool: age, sex, body weight, CYP2C19 phenotype, serum albumin, total bilirubin, serum creatinine, daily voriconazole dose, route of administration, rifampin-like CYP inducer co-administration, C-reactive protein (CRP; log-transformed as ln [CRP + 0.1]), days since the start of the current regimen, proton pump inhibitor use, and tacrolimus/calcineurin inhibitor use.

These variables were selected on pharmacological grounds. CRP captures inflammation-mediated suppression of hepatic CYP activity (Klomp et al., 2024). Proton pump inhibitors were included as potential CYP2C19 competitive inhibitors, and tacrolimus because its co-prescription in transplant recipients has clinical monitoring implications. Days on the current regimen was added to capture any time-dependent change in metabolic capacity. CRP availability defined the complete-case dataset used for both models.

Laboratory values were assigned to the TDM sample using the measurement closest to the TDM sample after manual review, with a preference for values obtained within ±24 h of the TDM sample.

### 2.6 Statistical analysis

Continuous variables are presented as median (interquartile range) and categorical variables as number (percentage); normality was assessed by the Shapiro–Wilk test and skewness, and all continuous variables except BMI were non-normal (Supplementary Table S1). *CYP2C19* same-platform baseline prevalence and Hardy–Weinberg analyses are summarised in Supplementary Table S2. Factors associated with subtherapeutic and supratherapeutic exposure were examined with Bayesian mixed-effects logistic regression, fitting separate models for the two outcomes on a shared covariate set with a patient-level random intercept to account for correlation among repeated measurements. This penalised approach was adopted because the subtherapeutic outcome was sparse relative to the covariate count and the rifampin-like inducer term was affected by near-complete separation, conditions under which maximum-likelihood estimates and their Wald intervals are unstable (Supplementary Table S3). Weakly informative priors were placed on the fixed effects (Student-t with three degrees of freedom, centred at zero, scale 2.5), on the intercept (Student-t, scale 10) and on the random-intercept standard deviation (half-normal, scale 1). Models were fitted by Hamiltonian Monte Carlo (the No-U-Turn sampler) with four chains of 1,000 warm-up and 1,000 retained draws each; convergence was confirmed by potential scale-reduction factors of at most 1.002, effective sample sizes above 1,900, and the absence of divergent transitions. Estimates are reported as posterior median odds ratios with 95% credible intervals, and an association was considered credible when its interval excluded unity. Because CRP was unavailable for some episodes, both models were fitted in a complete-case covariate set of 445 episodes from 139 patients; episodes with and without CRP were compared and the supratherapeutic model was refitted without CRP as a robustness check (Supplementary Tables S4, S5). As sensitivity analyses we refitted both models by maximum likelihood (Laplace approximation, bobyqa optimiser; Supplementary Table S6), confirmed robustness to a heavier-tailed Cauchy (0, 2.5) prior, and repeated the primary fit after excluding the two rapid-metaboliser patients. Further prespecified sensitivity analyses restricted the data to steady-state episodes and to precisely timed troughs (Supplementary Table S7). Adjacent TDM pairs with a documented dose change were analysed descriptively for category transitions; only a change in the numerical daily dose counted as a dose change, and where multiple changes occurred between adjacent troughs the net change was used (Supplementary Methods). Analyses used R version 4.3.2 with the brms package for the primary Bayesian models (Bürkner, 2017) and the lme4 package for the maximum-likelihood sensitivity analyses (Bates et al., 2015).

## 3 Results

### 3.1 Study population

**FIGURE 1:**
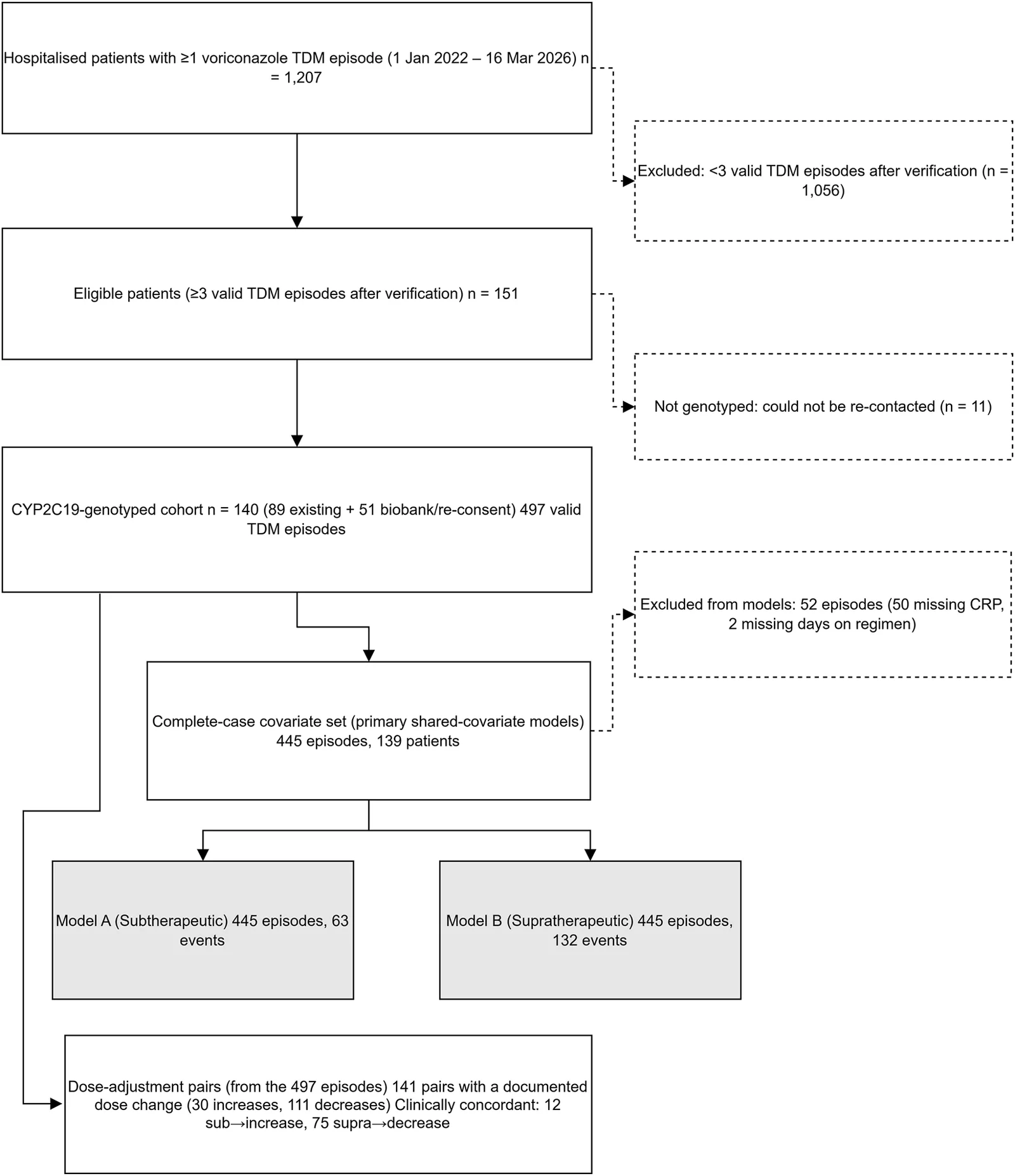
Flowchart of patient selection and analysis sets. Of 1,207 hospitalised patients who received voriconazole with at least one TDM measurement, 151 met the criterion of at least three valid episodes after verification; 11 without *CYP2C19* genotype results were excluded, leaving 140 patients contributing 497 episodes. The shared-covariate models were fitted in a complete-case dataset of 445 episodes from 139 patients (63 subtherapeutic and 132 supratherapeutic events). A total of 141 dose-adjustment pairs were identified.

Of 1,207 hospitalised patients who received voriconazole and had at least one TDM measurement during the study period, 151 met the criterion of at least three valid TDM episodes after verification. Eleven of these lacked *CYP2C19* genotype results and were excluded, leaving 140 patients contributing 497 valid TDM episodes (Figure 1). These 140 patients comprised 89 with existing clinical genotype records and 51 genotyped through biobank/re-consent procedures. The median number of episodes per patient was 3 (IQR 3–4; range 3–6).

In the shared-covariate models, the complete-case covariate set included 445 episodes from 139 patients; the 52 excluded episodes were mainly due to missing CRP (50 episodes), with two further episodes excluded because days since regimen start could not be confirmed. A total of 141 dose-adjustment pairs were identified (111 decreases, 30 increases).

The median age was 62.0 years (IQR 52.0–71.2), and 101 patients (72.1%) were male. Median body weight was 55.0 kg (IQR 48.3–64.6). *CYP2C19* phenotype distribution was 41 NM (29.3%), 2 RM (1.4%), 48 IM (34.3%), and 49 PM (35.0%; 49/140); the two rapid metabolisers carried the *1/*17 genotype. This poor-metaboliser proportion is higher than the approximately 13% reported for the general Han Chinese population (He et al., 2020). On the same genotyping platform, an unselected institutional comparison sample tested for non-voriconazole indications showed a poor-metaboliser prevalence of 18.6% (8/43) and was in Hardy–Weinberg equilibrium (P = 0.77), whereas the repeated-TDM cohort deviated from Hardy–Weinberg equilibrium (P = 0.0002) with a deficit of intermediate metabolisers, consistent with selection into repeated monitoring (Supplementary Table S2).

To characterise this selection directly, we compared baseline characteristics across patients contributing one, two, or three or more valid TDM episodes in a supplementary comparison. In that comparison, the ≥3-episode group is the 140-patient genotyped analytic cohort; 11 additional patients with ≥3 valid episodes lacked genotype data and were therefore not included in the analysed cohort. Patients monitored once were mostly within range at their first measurement (59% in range, 31% supratherapeutic), whereas those monitored twice, and those forming the study cohort, were more often supratherapeutic at baseline (42% and 41%, respectively). The cohort was modestly older (median 62 versus 55–57 years), while sex distribution and departmental case-mix differed little. Selection into repeated monitoring was therefore driven mainly by out-of-range, and particularly supratherapeutic, exposure rather than by demography, so the cohort should be read as the repeated-monitoring population rather than as representative of all voriconazole recipients.

Thirteen patients (9.3%) had chronic liver disease. Patients came mainly from respiratory medicine (34.3%), transplant services (10.0%), hepatic disease services (8.6%), HIV/immunodeficiency care (6.4%), intensive care (20.7%), and other departments (20.0%). At the episode level, 364 episodes (73.2%) involved oral administration. Median daily dose was 400 mg (IQR 200–400), albumin 34.7 g/L (IQR 32.0–37.6), total bilirubin 9.9 μmol/L (5.7–19.2), serum creatinine 72.0 μmol/L (55.0–107.0), and CRP 25.0 mg/L (9.4–61.1; available in 447 of 497 episodes). PPIs were present in 10.9% of episodes, rifampin-like inducers in 2.4%, and tacrolimus/calcineurin inhibitors in 15.3% (Table 1).

**TABLE 1:** Baseline characteristics of the study population.

Characteristic	Value
Patient-level characteristics (n = 140)	
Age, years, median (IQR)	62.0 (52.0–71.2)
Male, n (%)	101 (72.1)
Body weight, kg, median (IQR)	55.0 (48.3–64.6)
BMI, kg/m^2^, median (IQR)	20.7 (18.1–23.1)
*CYP2C19* phenotype, n (%)	
NM	41 (29.3)
RM	2 (1.4)
IM	48 (34.3)
PM	49 (35.0)
Chronic liver disease, n (%)	13 (9.3)
Department, n (%)	
Respiratory	48 (34.3)
ICU/Critical care	29 (20.7)
Transplant	14 (10.0)
Hepatic disease	12 (8.6)
HIV/Immunodeficiency	9 (6.4)
Other	28 (20.0)
TDM episode-level characteristics (n = 497)	
TDM episodes per patient, median (IQR; range)	3 (3–4; 3–6)
Route: oral, n (%)	364 (73.2)
Daily dose, mg, median (IQR)	400 (200–400)
Albumin, g/L, median (IQR)	34.7 (32.0–37.6)
Total bilirubin, µmol/L, median (IQR)	9.9 (5.7–19.2)
Serum creatinine, µmol/L, median (IQR)	72.0 (55.0–107.0)
CRP, mg/L, median (IQR)^a^	25.0 (9.4–61.1)
PPI use, n (%)	54 (10.9)
Rifampin-like inducer, n (%)	12 (2.4)
Tacrolimus/CNI use, n (%)	76 (15.3)
Antiretroviral agent, n (%)	22 (4.4)

^a^ CRP, available for 447 of 497 episodes (89.9%). BMI, body mass index; CRP, C-reactive protein; CNI, calcineurin inhibitor; HIV, human immunodeficiency virus; ICU, intensive care unit; IM, intermediate metaboliser; IQR, interquartile range; NM, normal metaboliser; PM, poor metaboliser; RM, rapid metaboliser; PPI, proton pump inhibitor; TDM, therapeutic drug monitoring.

### 3.2 Distribution of voriconazole exposure

Median trough concentration was 3.1 μg/mL (IQR 1.5–5.5). Of the 497 episodes, 72 (14.5%) were subtherapeutic, 280 (56.3%) were in range, and 145 (29.2%) were supratherapeutic. Thus, almost 44% of monitored episodes remained outside the conventional target range despite repeated monitoring.

The distribution differed by *CYP2C19* phenotype. Supratherapeutic exposure occurred in 79 of 169 PM episodes (46.7%), compared with 30 of 144 NM episodes (20.8%) and 35 of 178 IM episodes (19.7%), and 1 of 6 rapid-metaboliser episodes. Among the 12 episodes with rifampin-like inducer co-administration, 7 (58.3%) were subtherapeutic.

### 3.3 Factors associated with subtherapeutic and supratherapeutic exposure

**FIGURE 2:**
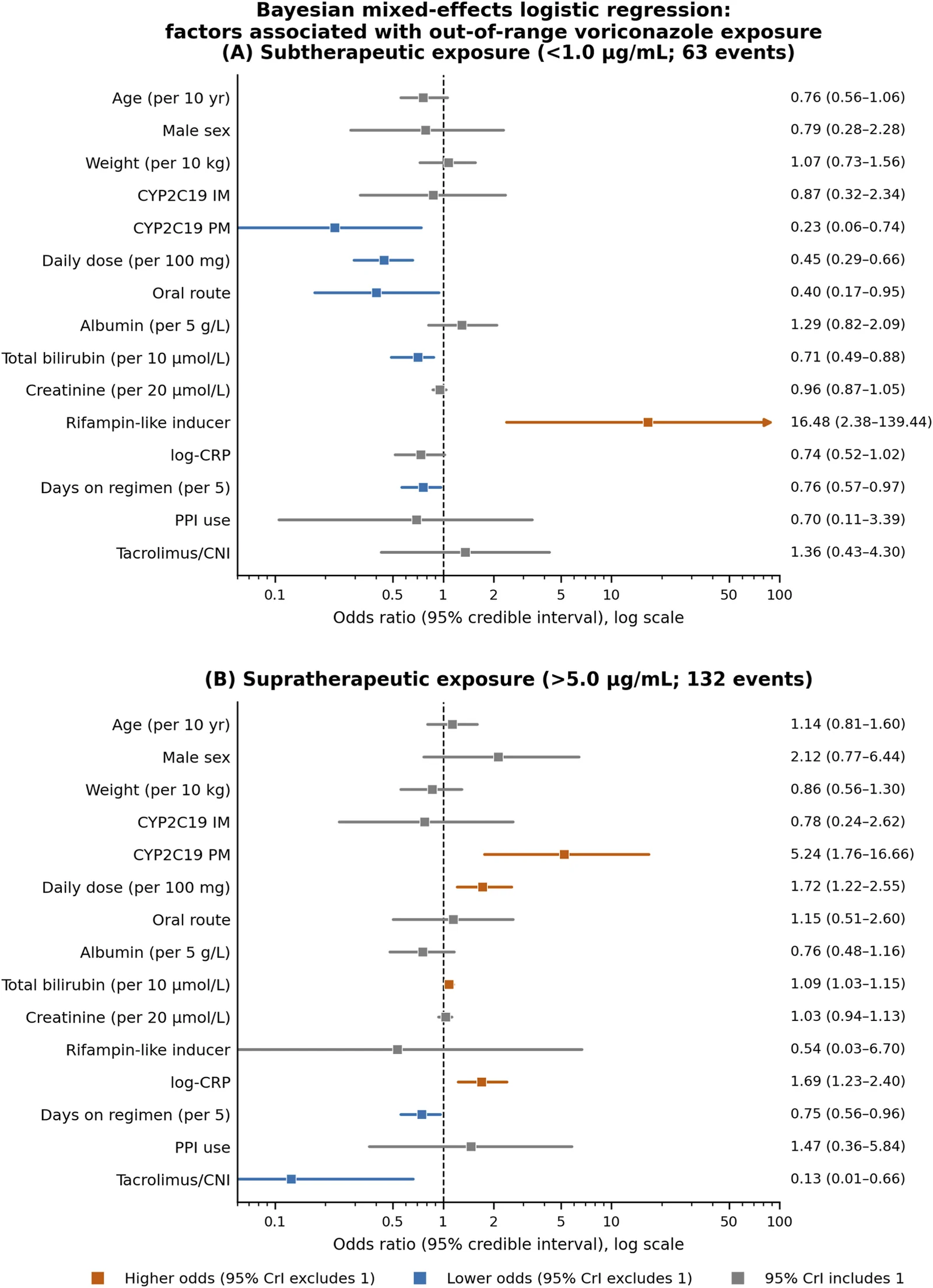
Forest plot of Bayesian mixed-effects logistic regression results. Both models were fitted on the same 445-episode complete-case dataset with an identical 15-covariate specification and a patient-level random intercept. **(A)** Factors associated with subtherapeutic exposure (<1.0 μg/mL; 63 events). **(B)** Factors associated with supratherapeutic exposure (>5.0 μg/mL; 132 events). Points are posterior median odds ratios and bars are 95% credible intervals, shown on a logarithmic scale; the upper limit of the rifampin-like inducer interval in panel A extends beyond the axis and is marked with an arrow.

In Model A (445 episodes, 63 subtherapeutic events), rifampin-like CYP inducer co-administration showed the strongest association with subtherapeutic risk (OR 16.48, 95% CrI 2.38–139.44); the wide interval reflects the small number of exposed episodes, but its lower bound remained well above unity. Lower daily dose was the other main contributor (OR 0.45 per 100 mg, 95% CrI 0.29–0.66). Oral administration was associated with lower odds of subtherapeutic exposure than intravenous administration (OR 0.40, 95% CrI 0.17–0.95), as were the PM phenotype (OR 0.23, 95% CrI 0.06–0.74), higher total bilirubin (OR 0.71 per 10 μmol/L, 95% CrI 0.49–0.88) and longer time on the current regimen (OR 0.76 per 5 days, 95% CrI 0.57–0.97). Older age (OR 0.76 per 10 years, 95% CrI 0.56–1.06) and log-CRP (OR 0.74, 95% CrI 0.52–1.02) showed inverse associations whose credible intervals included unity (Table 2; Figure 2).

**TABLE 2:** Bayesian mixed-effects logistic regression results for out-of-range voriconazole exposure.

Variable	Model A: Subtherapeutic exposure (<1.0 μg/mL)		Model B: Supratherapeutic exposure (>5.0 μg/mL)	
	OR	95% CrI	OR	95% CrI
Age (per 10 yr)	0.76	0.56–1.06	1.14	0.81–1.60
Male sex	0.79	0.28–2.28	2.12	0.77–6.44
Weight (per 10 kg)	1.07	0.73–1.56	0.86	0.56–1.30
*CYP2C19* IM vs NM	0.87	0.32–2.34	0.78	0.24–2.62
*CYP2C19* PM vs NM	0.23	0.06–0.74	5.24	1.76–16.66
Daily dose (per 100 mg)	0.45	0.29–0.66	1.72	1.22–2.55
Oral vs IV	0.40	0.17–0.95	1.15	0.51–2.60
Albumin (per 5 g/L)	1.29	0.82–2.09	0.76	0.48–1.16
Total bilirubin (per 10 µmol/L)	0.71	0.49–0.88	1.09	1.03–1.15
SCr (per 20 µmol/L)	0.96	0.87–1.05	1.03	0.94–1.13
Rifampin-like inducer	16.48	2.38–139.44	0.54	0.03–6.70
CRP (log)	0.74	0.52–1.02	1.69	1.23–2.40
Days on regimen (per 5)	0.76	0.57–0.97	0.75	0.56–0.96
PPI use	0.70	0.11–3.39	1.47	0.36–5.84
Tacrolimus/CNI	1.36	0.43–4.30	0.13	0.01–0.66

Model A: subtherapeutic (<1.0 μg/mL), n = 445 episodes, 63 events. Model B: supratherapeutic (>5.0 μg/mL), n = 445 episodes, 132 events. Both models were fitted on the same complete-case covariate set with an identical 15-covariate specification. Values are posterior median odds ratios with 95% credible intervals from Bayesian mixed-effects logistic regression with patient-level random intercepts and weakly informative priors; an association was considered credible when its interval excluded 1. The maximum-likelihood fit of the same models is provided as a sensitivity analysis in Supplementary Table S6. CrI, credible interval; CNI, calcineurin inhibitor; CRP, C-reactive protein; IV, intravenous; IM, intermediate metaboliser; NM, normal metaboliser; OR, odds ratio; PM, poor metaboliser; PPI, proton pump inhibitor; SCr, serum creatinine.

In Model B (445 episodes, 132 supratherapeutic events), the PM phenotype carried the strongest association (OR 5.24, 95% CrI 1.76–16.66). Higher log-CRP (OR 1.69, 95% CrI 1.23–2.40), higher total bilirubin (OR 1.09 per 10 μmol/L, 95% CrI 1.03–1.15), and higher daily dose (OR 1.72 per 100 mg, 95% CrI 1.22–2.55) were also associated. Tacrolimus/calcineurin inhibitor co-administration was inversely associated with supratherapeutic exposure (OR 0.13, 95% CrI 0.01–0.66), as was longer treatment duration (OR 0.75 per 5 days, 95% CrI 0.56–0.96) (Table 2; Figure 2). The corresponding shared-covariate estimates for both outcomes are also listed in Supplementary Table S6.

### 3.4 Return to target range after dose adjustment

Clinically concordant dose-adjustment pairs showed a clear asymmetry (Table 3). Among the 12 pairs in which a subtherapeutic concentration was followed by dose increase, 7 of 12 (58.3%) returned to range, 4 of 12 (33.3%) remained subtherapeutic, and 1 of 12 (8.3%) became supratherapeutic.

**TABLE 3:** Concentration category transitions after clinically concordant dose adjustment.

Pre-adjustment category	Dose change	n	Returned to range	Remained/became sub	Remained/became supra
Subtherapeutic	Increase	12	7 (58.3)	4 (33.3)	1 (8.3)
Supratherapeutic	Decrease	75	26 (34.7)	8 (10.7)	41 (54.7)

Values are n (row %). Clinically concordant pairs: subtherapeutic episodes followed by dose increase and supratherapeutic episodes followed by dose decrease. Returned to range = post-adjustment concentration 1.0–5.0 μg/mL. sub, subtherapeutic (<1.0 μg/mL); supra, supratherapeutic (>5.0 μg/mL). The 75 dose reductions were contributed by 55 patients; because these observations are not independent, cell percentages should not be interpreted as patient-level response rates. The full transition matrix including all dose-adjustment pairs is provided in Supplementary Table S10.

Among the 75 pairs in which a supratherapeutic concentration was followed by dose decrease, 26 of 75 (34.7%) returned to range, 8 of 75 (10.7%) became subtherapeutic, and 41 of 75 (54.7%) remained supratherapeutic; thus, more than half of supratherapeutic episodes remained above target after dose reduction. These 75 reductions were contributed by 55 patients, so the observations are not independent and the stratum percentages should not be read as patient-level response rates. Characterisation of the reductions (Supplementary Tables S8, S9) showed that the concentration response was variable and frequently sub-proportional to the dose change, that 40% of re-measurements preceded a new steady state, and that re-testing within 7 days was associated with a lower apparent normalisation rate (27% versus 48%); larger reductions were not clearly associated with a higher likelihood of normalisation. The full transition matrix for all 141 pairs is shown in Supplementary Table S10.

## 4 Discussion

In this repeated-TDM cohort, out-of-range voriconazole exposure remained common despite continued monitoring: almost 44% of episodes were below or above the conventional target range. The two directions of risk were not associated with the same pattern of factors. Low concentrations were mainly associated with lower dose and rifampin-like induction, whereas high concentrations were associated with poor-metaboliser status, inflammation, bilirubin and higher dose. Most of these individual associations are confirmatory of established voriconazole pharmacology; the value of this analysis lies in describing how they play out across repeated monitoring and dose adjustment in routine inpatient care. Dose adjustment pointed in the same direction: increasing the dose after a low concentration was more often followed by return to range than reducing the dose after a high concentration. This is consistent with a meta-analysis of 60 studies reporting a pooled therapeutic-range attainment of 56% without dose adjustment (Li et al., 2024).

Subtherapeutic exposure was most strongly associated with lower daily dose and rifampin-like inducer co-administration, consistent with inadequate drug input or increased clearance. The strong association with rifampin-like inducers reflects the well-known capacity of rifamycins to reduce voriconazole exposure, and such combinations should generally be avoided (Geist et al., 2007; Lu et al., 2024). Oral administration was associated with lower subtherapeutic risk than intravenous dosing, which may reflect clinical practice that reserves intravenous voriconazole for more acutely ill patients in whom absorption is less reliable or effective doses are not yet established. Clinicians encountering repeated subtherapeutic concentrations should consider concurrent enzyme inducers, the adequacy of the prescribed dose and the route of administration as the most likely explanations.

By contrast, supratherapeutic exposure was associated with *CYP2C19* PM status, elevated CRP and higher total bilirubin—all factors that reduce metabolic capacity through different mechanisms. The link between PM phenotype and supratherapeutic concentration is well recognised (Chen et al., 2022) and is unsurprising given the dominant role of CYP2C19 in voriconazole metabolism; *CYP2C19* genotype-guided dosing has been shown to reduce the composite incidence of hepatotoxicity and visual symptoms without compromising response (Katada et al., 2025). PMs comprised 35.0% of this cohort, higher than the approximately 13% reported in the general Han Chinese population (He et al., 2020). Such enrichment is expected in a repeated-TDM cohort: patients at the metabolic extremes are more likely to have persistently out-of-range concentrations and therefore accumulate more monitoring episodes—a form of selection bias to keep in mind when interpreting such data. Consistent with this, the same genotyping platform applied to an unselected non-voriconazole sample gave a poor-metaboliser prevalence of 18.6% and was in Hardy–Weinberg equilibrium, whereas the present cohort departs from equilibrium through a deficit of intermediate metabolisers and an excess at both metabolic extremes—the pattern expected when metabolic outliers are preferentially selected into repeated monitoring (Supplementary Table S2). A systematic genotyping error is therefore unlikely, and the prevalence is not generalised to all patients receiving voriconazole.

An independent association between log-CRP and supratherapeutic exposure is consistent with literature indicating that inflammation inhibits hepatic CYP activity, reducing voriconazole clearance and raising concentrations (van Wanrooy et al., 2014). Proinflammatory cytokines, especially interleukin-6, downregulate CYP2C19 in a process termed inflammation-driven phenoconversion (Veringa et al., 2017; Bolcato et al., 2021; Klomp et al., 2024). Klomp and colleagues showed that moderate-to-severe inflammation (CRP >50 mg/L) effectively phenocopies patients into a lower metaboliser category regardless of genotype, with dose-corrected concentrations increasing by 245%–486% (Klomp et al., 2024); a recent review identified CRP, *CYP2C19* genotype, age and dose as the primary covariates affecting metabolic ratio and N-oxide exposure (Hubrechts et al., 2026). With a median CRP of 25.0 mg/L and many values >60 mg/L, inflammation-mediated reduction in clearance may have contributed to supratherapeutic exposure among PM and IM patients. Whether genotype and inflammation act additively or synergistically cannot be determined here, but both commonly coexist in acutely ill inpatients. An elevated CRP should raise suspicion that a measured concentration reflects reduced clearance rather than excessive dosing, and dose reduction alone may be insufficient if the inflammatory trigger is unresolved.

Total bilirubin was also associated with supratherapeutic exposure, an additional hepatic signal of impaired clearance, notable given voriconazole’s hepatotoxic potential and the possibility of a vicious cycle in which liver injury further impairs clearance (Hu et al., 2024). Tacrolimus/calcineurin inhibitor co-administration was inversely associated with supratherapeutic exposure, but this likely reflects management-related confounding rather than a true pharmacokinetic effect: transplant patients on interacting therapies are often monitored more closely and dose-adjusted more proactively (Zhao et al., 2022). A predictive model for voriconazole-related liver injury identified trough concentration and hypoproteinaemia as independent risk factors (Xiao et al., 2023), reinforcing the clinical relevance of the vicious-cycle hypothesis.

That supratherapeutic concentrations frequently did not return to range after dose reduction is a descriptive observation, and several non-exclusive explanations are plausible. The analysis was unadjusted and the reductions were not independent, so confounding by indication and regression to the mean are difficult to exclude: the most extreme concentrations were both the most likely to prompt a large reduction and the most likely to fall on retesting irrespective of any dose change. Sampling time is a further consideration, because about 40% of re-measurements preceded a new steady state and would tend to understate the eventual decline. Voriconazole’s saturable, nonlinear metabolism offers a pharmacological mechanism that could also contribute (Mangal et al., 2018; Li et al., 2020), as physiologically based modelling has shown time-dependent autoinhibition of CYP3A4 (Li et al., 2020); once metabolising enzymes are saturated a dose reduction may yield only a slow decline, and this window is plausibly narrower when supratherapeutic exposure reflects a poor-metaboliser genotype, active inflammation or hepatic dysfunction. These explanations cannot be separated in the present data. Whatever their relative contributions, the practical implication is the same: clinicians should anticipate that a single dose reduction may be insufficient and should schedule confirmatory TDM once a new steady state is reached.

Several strengths should be noted. Patient records were manually verified, and the analysis focused on repeated monitoring rather than single time-point exposure. Separating subtherapeutic and supratherapeutic risk also provided a direction-specific view beyond overall target attainment alone. Limitations should also be acknowledged. The design was retrospective and single-centre, and residual confounding cannot be excluded. A repeated-TDM cohort is a selected inpatient subgroup and should not be generalised to all patients receiving voriconazole; the cohort comprised only adults. Because dosing decisions in routine care respond to previous concentrations, daily dose is not an exogenous exposure; the random intercept accounts for within-patient correlation but not this treatment-by-indication feedback, so the dose associations should be read as descriptive of practice rather than causal, and disentangling pharmacokinetic determinants from dosing responses would require longitudinal causal methods that this cohort cannot support. The antifungal indication—prophylaxis versus treatment, and a proven/probable/possible classification—was not consistently recorded. CRP was unavailable in 10.1% of episodes, restricting Model B to a complete-case dataset. Finally, the dose-adjustment analysis was descriptive: it shows what happened after clinician-directed adjustments in practice, not what would have happened under a controlled dosing strategy.

## 5 Conclusion

In hospitalised patients undergoing repeated voriconazole TDM, out-of-range exposure was common, and the factors associated with subtherapeutic and supratherapeutic concentrations differed by direction and were clinically interpretable. Subtherapeutic exposure was associated with lower daily dose and rifampin-like enzyme induction, whereas supratherapeutic exposure reflected *CYP2C19* poor-metaboliser status, elevated CRP, and higher total bilirubin. After dose reduction, supratherapeutic concentrations frequently did not return to the therapeutic range; this descriptive pattern is compatible with several contributors, including resampling before a new steady state, confounding by indication and voriconazole’s nonlinear metabolism. External validation in other populations and settings is needed.

## Data Availability

The datasets presented in this article are not readily available due to privacy and ethical restrictions. Requests to access the datasets should be directed to Wei Li (8333628@qq.com).
