# Supplementary material for: Repeated voriconazole therapeutic drug monitoring in hospitalised patients: a real-world cohort study of out-of-range exposure and concentration response to dose adjustment
Source: Front Pharmacol. 2026 Sep 2;17:1867435. doi: 10.3389/fphar.2026.1867435 (PMC13579564; doi:10.3389/fphar.2026.1867435)
Supplement: Supplementary file 1 [file DataSheet1.pdf]

Supplementary Material

1 Supplementary Methods

1.1 Trough definition

A blood sample was considered a trough if drawn within approximately one hour before the next scheduled voriconazole dose, or during early-morning phlebotomy (06:00–07:30) in patients receiving stable twice-daily regimens. Samples with known non-trough timing or ambiguous sampling context were excluded during data verification.

1.2 Valid TDM episode criteria

A TDM episode was valid if all of the following held: the sample was clinically interpretable as a trough; the preceding voriconazole regimen (dose, frequency and route) was identifiable from structured medication records; and the assay result could be standardised to μg/mL. No minimum number of prior doses was required, but a steady-state flag was recorded for each episode.

1.3 Below-limit-of-quantification handling

Concentrations below the lower limit of quantification (0.48 μg/mL for HPLC; 0.05 μg/mL for LC-MS/MS) were retained with a flag and classified as subtherapeutic (<1.0 μg/mL) in the categorical analyses; the laboratory-reported censored value was retained as the recorded concentration.

1.4 Laboratory covariate time window

Albumin, total bilirubin, serum creatinine and CRP were taken as the nearest measurements within ±24 hours of each TDM sample; where several fell within the window, the closest in time was used. If none was available within ±24 hours, the most proximate clinically relevant result was used after manual review.

1.5 CRP availability

CRP was available for 447 of 497 episodes (89.9%). The 50 missing values came mainly from wards where CRP was not routinely ordered. Because both models included CRP and days since regimen start, two further episodes with CRP but without days-since-regimen-start were also excluded, giving a complete-case covariate set of 445 episodes from 139 patients.

1.6 CYP2C19 phenotype assignment

Phenotype was assigned from structured genotype data per the CPIC guideline for CYP2C19 and voriconazole. Genotyping used a fluorescence-hybridisation assay detecting the **2* (c.681G>A), **3* (c.636G>A) and **17* (c.-806C>T) alleles. Patients with **1/*1* were classified as normal metabolisers (NM); **1/*2* and **1/*3* as intermediate metabolisers (IM); and **2/*2, *2/*3* and **3/*3* as poor metabolisers (PM). Two patients carrying **1/*17* were classified as rapid metabolisers (RM) and shown separately in Table 1; owing to their small number they were retained within the normal-metaboliser reference category for the regression models. Excluding these two patients from the Bayesian model did not materially change the key subtherapeutic-model point estimates (posterior median OR 0.24 vs 0.23 for CYP2C19 PM vs NM; 15.5 vs 16.48 for rifampin-like inducer). Genotype results were obtained retrospectively from clinical records and the institutional biobank; of

151 patients meeting the monitoring criteria, 140 (92.7%) had genotype results and were included. Because alleles beyond **2, *3* and **17* were not assessed, rapid/ultrarapid misclassification cannot be fully excluded, although this is most relevant to fast-metaboliser classification and less so to the poor-metaboliser findings emphasised here.

1.7 Concentration categories

Trough concentrations were classified as subtherapeutic (<1.0 μg/mL), in range (1.0–5.0 μg/mL), or supratherapeutic (>5.0 μg/mL), based on commonly used target ranges for invasive fungal infections.

1.8 Dose-adjustment pairs definition

A dose-adjustment pair was two adjacent valid TDM episodes from the same patient with a documented change in daily voriconazole dose between them. Where more than one change occurred between two adjacent troughs, the pair reflected the net change between the dose in effect at the earlier trough and that at the later trough. Only a change in the numerical daily dose counted as a dose change; temporary withholding and discontinuation (a daily dose of zero) did not form pairs, and a change of route at an unchanged daily dose was not a dose change (route was modelled separately). A total of 141 pairs were identified (30 increases, 111 decreases); clinically concordant pairs were subtherapeutic episodes followed by dose increase (n = 12) and supratherapeutic episodes followed by dose decrease (n = 75).

1.9 Steady-state and precise-trough flags

Two quality flags were recorded for each episode. A trough was flagged as precisely timed when the documented sampling time fell within approximately one hour before the next scheduled dose, as distinct from an early-morning phlebotomy trough. An episode was flagged as being at steady state when sampled at least three days into an unchanged maintenance regimen, by which time voriconazole approaches steady state; episodes sampled earlier, or shortly after a dose change, were flagged as non-steady-state. Loading-dose and missed-dose episodes were identifiable from the medication record and were not treated as routine troughs.

1.10 Missing-data handling

Both models were fitted in the complete-case covariate set (445 episodes). Episodes with and without CRP were compared (Table S4), and the supratherapeutic model was refitted without CRP using all available episodes (Table S5) to assess robustness. CRP, the principal inflammatory exposure, was not imputed.

1.11 Model fitting, stability and diagnostics

Bayesian mixed-effects logistic regression with weakly informative priors was used for the Bayesian models; the maximum-likelihood fits summarised here are presented as sensitivity analyses and document the sparse-data conditions that motivated the Bayesian approach. The maximum-likelihood models were fitted by Laplace approximation with the bobyqa optimiser; both converged without warnings. Random-intercept variances were 0.40 (Model A) and 1.71 (Model B), corresponding to intraclass correlations of 0.11 and 0.34, with 4.2 and 8.8 events per variable in the shared-covariate models (Table S3). The rifampin-like inducer term in Model A was affected by near-complete separation (12 exposed episodes) and was corroborated by a simplified model, a profile-likelihood interval and an unadjusted exact test (Table S3). Posterior inferences were also materially unchanged under a heavier-tailed Cauchy (0, 2.5) prior.

1.12 Sensitivity analyses

Supplementary Table S6 presents the maximum-likelihood shared-covariate analysis of both outcomes on the identical complete-case set with identical covariates; these estimates provide a maximum-likelihood sensitivity analysis for the Bayesian models shown in Table 2. Additional sensitivity analyses comprised the with/without-CRP comparison (Table S5); and analyses restricted to steady-state episodes and to precisely timed troughs (Table S7).

1.13 Statistical software

Analyses were performed in R version 4.3.2 using the brms package for the Bayesian models and the lme4 package for the maximum-likelihood sensitivity analyses.

2 Supplementary Tables

Supplementary Table S1. Distribution of continuous variables

Variable	Median (IQR)	Skewness	Distribution
Age, years	62.0 (52.0–71.2)	−0.52	Non-normal
Body weight, kg	55.0 (48.3–64.6)	+0.66	Non-normal
BMI, kg/m²	20.7 (18.1–23.1)	+0.33	Normal
Serum albumin, g/L	34.7 (32.0–37.6)	+0.29	Non-normal
Total bilirubin, μmol/L	9.9 (5.7–19.2)	+3.47	Non-normal
Serum creatinine, μmol/L	72.0 (55.0–107.0)	+4.17	Non-normal
CRP, mg/L	25.0 (9.4–61.1)	+1.96	Non-normal
Daily dose, mg	400 (200–400)	+0.16	Non-normal
Trough concentration, μg/mL	3.1 (1.5–5.5)	+1.66	Non-normal

Normality assessed by the Shapiro–Wilk test and skewness. All continuous variables except BMI were non-normal; variables are therefore summarised as median (IQR).

Supplementary Table S2. CYP2C19 poor-metaboliser prevalence: same-platform baseline and Hardy–Weinberg analysis

S2-1. Poor-metaboliser prevalence across reference samples

Sample (same platform unless noted)	Indication	n	PM n	PM %
National pooled Han Chinese (He et al., 2020)	—	>3200	—	~13%
Institutional comparison sample	Non-voriconazole (antiplatelet)	43	8	18.6%
Voriconazole repeated-TDM	Voriconazole TDM	140	49	35.0%

cohort (this study)				

National figure from He et al. (2020). PM, poor metaboliser.

S2-2. Hardy–Weinberg analysis (functional [*1, *17] vs reduced-function [*2, *3] alleles)

Measure	Institutional (n = 43)	Cohort (n = 140)
Reduced-function allele frequency	0.42	0.52
NM (0): observed / expected	15 / 14.5	43 / 32.1
IM (1, heterozygous): observed / expected	20 / 20.9	48 / 69.9
PM (2): observed / expected	8 / 7.5	49 / 38.1
Hardy–Weinberg χ^2 (df = 1)	0.09	13.72
Hardy–Weinberg P	0.77	0.0002

Cohort genotypes: **1/*1* 41, **1/*2* 47, **1/*3* 1, **2/*2* 40, **2/*3* 9, **1/*17* 2. *Institutional genotypes: *1/*1* 15, **1/*2* 15, **1/*3* 5, **2/*2* 5, **2/*3* 3. The unselected institutional sample is in Hardy–Weinberg equilibrium with heterozygotes at the expected frequency, excluding systematic genotyping error; the cohort deviates through a deficit of intermediate metabolisers and excess at both extremes, the expected signature of selection into repeated monitoring. Of 151 eligible patients, 140 (92.7%) were genotyped; the worst-case bound on cohort PM prevalence is 32.5–39.7%.

Supplementary Table S3. Model diagnostics and rifampin-like inducer sensitivity

S3-1. Random-intercept model diagnostics

Model	Random-intercept variance	Intraclass correlation	Events per variable
A (subtherapeutic)	0.40	0.11	4.2
B (supratherapeutic)	1.71	0.34	8.8

Both models converged without warnings (Laplace approximation, bobyqa optimiser).

S3-2. Rifampin-like inducer association under different estimators (Model A)

Estimator	OR (95% CI) for subtherapeutic exposure
Full mixed-effects model (Wald)	17.99 (3.11–104.15)
Simplified mixed-effects model	16.38 (3.41–78.62)
Profile-likelihood interval	18.56 (3.98–109.51)
Unadjusted exact test (Fisher)	8.98 (2.37–36.99)

Twelve episodes involved rifampin-like inducer exposure (7 of 12 subtherapeutic versus 65 of 485 unexposed), producing near-complete separation. The association is large and directionally robust across estimators, but its magnitude is imprecise.

Supplementary Table S4. Episodes with versus without CRP

Characteristic	CRP available (n = 447)	CRP missing (n = 50)	P
Supratherapeutic, %	30	26	0.74
Subtherapeutic, %	14	16	0.68

Oral route, %	72	86	0.042
CYP2C19 PM, %	33	40	0.35
Daily dose, mg (median)	400	300	0.11
Total bilirubin, µmol/L (median)	10	13	0.84
Age, years (median)	62	58	0.083

Fisher exact test (categorical) and Mann–Whitney test (continuous). Missingness was unrelated to the outcome and was structured by ward (routine CRP ordering varied by department), consistent with a missing-at-random pattern.

Supplementary Table S5. Supratherapeutic model with and without CRP

Variable	With CRP (445 ep, 132 ev)	Without CRP (494 ep, 145 ev)
CYP2C19 PM vs NM	4.65 (1.69–12.81)*	4.79 (1.88–12.18)*
Daily dose (per 100 mg)	1.58 (1.13–2.20)*	1.52 (1.12–2.07)*
Bilirubin (per 10 µmol/L)	1.08 (1.03–1.13)*	1.06 (1.02–1.10)*
Tacrolimus/CNI	0.14 (0.03–0.71)*	0.10 (0.03–0.41)*
Days on regimen (per 5)	0.78 (0.61–1.00)*	0.81 (0.67–0.97)*
Log-CRP	1.59 (1.18–2.14)*	—

Odds ratios (95% CI). The non-CRP associations are essentially unchanged whether or not CRP is in the model and whether or not the 50 CRP-missing episodes are included. * $P < 0.05$.

Supplementary Table S6. Shared covariate-set sensitivity analysis

Variable	Subtherapeutic (445 ep, 63 ev)	Supratherapeutic (445 ep, 132 ev)
Age (per 10 yr)	0.77 (0.59–1.00)*	1.11 (0.84–1.47)
Male sex	0.81 (0.33–1.95)	2.06 (0.81–5.24)
Weight (per 10 kg)	1.09 (0.80–1.47)	0.87 (0.62–1.23)
CYP2C19 IM vs NM	0.88 (0.38–2.05)	0.85 (0.31–2.36)
CYP2C19 PM vs NM	0.27 (0.09–0.77)*	4.65 (1.69–12.81)*
Daily dose (per 100 mg)	0.46 (0.32–0.68)*	1.58 (1.13–2.20)*
Oral vs IV	0.40 (0.18–0.85)*	1.20 (0.58–2.50)
Albumin (per 5 g/L)	1.27 (0.83–1.94)	0.78 (0.52–1.18)
Bilirubin (per 10 µmol/L)	0.76 (0.59–0.97)*	1.08 (1.03–1.13)*
SCr (per 20 µmol/L)	0.96 (0.89–1.04)	1.03 (0.95–1.11)
Rifampin-like inducer	17.99 (3.11–104.15)*	0.54 (0.04–6.87)
Log-CRP	0.79 (0.59–1.04)	1.59 (1.18–2.14)*
Days on regimen (per 5)	0.80 (0.63–1.01)	0.78 (0.61–1.00)*
PPI use	0.84 (0.18–3.93)	1.47 (0.43–5.05)
Tacrolimus/CNI	1.38 (0.53–3.64)	0.14 (0.03–0.71)*

Both outcomes were fitted on the identical complete-case set (445 episodes) with the identical full covariate set, so the only difference is the modelled outcome. The directional contrast held for the principal factors, including inverse versus positive CRP associations for subtherapeutic and supratherapeutic exposure. * P < 0.05.

Supplementary Table S7. Sensitivity analysis restricted to steady-state episodes and precisely timed troughs

Variable	Shared-covariate model	Steady-state subset
Supratherapeutic model		
CYP2C19 PM vs NM	4.65 (1.69–12.81)	4.76 (1.04–21.71)*
Log-CRP	1.59 (1.18–2.14)	1.67 (1.05–2.66)*
Bilirubin (per 10 µmol/L)	1.08 (1.03–1.13)	1.12 (1.04–1.21)*
Daily dose (per 100 mg)	1.58 (1.13–2.20)	2.00 (1.11–3.61)*
Tacrolimus/CNI	0.14 (0.03–0.71)	0.22 (0.03–1.80)
Subtherapeutic model		
Daily dose (per 100 mg)	0.46 (0.32–0.68)	0.49 (0.32–0.76)*
CYP2C19 PM vs NM	0.27 (0.09–0.77)	0.30 (0.10–0.91)*
Oral vs IV	0.40 (0.18–0.85)	0.25 (0.10–0.60)*
Age (per 10 yr)	0.77 (0.59–1.00)	0.72 (0.55–0.95)*

Supratherapeutic model: full covariate set, complete-case within the steady-state subset (261 episodes, 64 events). Subtherapeutic model: the corresponding shared-covariate maximum-likelihood estimates are shown in the first column; within the steady-state subset, a simplified core-covariate model (303 episodes, 39 events) was fitted with the Nelder–Mead optimiser (random-intercept variance 0.39) because the default optimiser is near-singular at this reduced event count; the rifampin-like inducer term was not re-estimated because few exposed episodes were at steady state. A parallel restriction to precisely-timed troughs (316 episodes) gave concordant point estimates. * P < 0.05 in the subset.

Supplementary Table S8. Characterisation of the 75 concordant dose reductions

Characteristic (75 reductions, 55 patients)	Value
Pre-adjustment trough, µg/mL, median (IQR)	7.5 (6.2–10.2)
Absolute daily-dose reduction, mg/day, median	100
Relative dose reduction, %, median (IQR)	33 (25–50)
Relative change in trough, %, median (IQR)	−33 (−78 to +1)
Sub-proportional response (trough fell less than dose), n (%)	36 (48)
Re-measurement interval, days, median (IQR)	6 (4–8)
At steady state at re-measurement, n (%)	45 (60)
Route switch between episodes, n (%)	6 (8)
Interim CRP change, mg/L, median (fell in 62%)	−3.8

Post-reduction trough, µg/mL, median (IQR)	5.5 (2.1–7.5)
Returned to range, n (%)	26 (35)
Remained supratherapeutic, n (%)	41 (55)
Fell subtherapeutic, n (%)	8 (11)
CYP2C19 PM / IM / NM, n	42 / 16 / 17

Negative values for change in trough indicate a decrease. Descriptive summary only.

Supplementary Table S9. Return to range after dose reduction, by stratum

Stratum (of 75 reductions)	Returned to range, %
Pre-adjustment trough 5–7 µg/mL	43
Pre-adjustment trough >7 µg/mL	30
Relative dose reduction <33%	37
Relative dose reduction ≥33%	33
Relative dose reduction <50%	41
Relative dose reduction ≥50%	27
Re-measurement <7 days	27
Re-measurement ≥7 days	48
CYP2C19 poor metaboliser	36
CYP2C19 non-poor metaboliser	33
Interim CRP fell	35
Interim CRP rose	24

Descriptive proportions among the 75 concordant reductions; no inferential testing was performed given the small, patient-overlapping sample. Larger reductions were not clearly associated with a higher normalisation rate, and re-measurement within seven days was associated with a lower apparent normalisation rate, suggesting that part of this pattern may reflect premature sampling.

Supplementary Table S10. Full concentration-category transition matrix after dose adjustment

Panel A. Dose decrease (n = 111)

Pre-adjustment	Post <1.0	Post 1.0–5.0	Post >5.0	Total
Subtherapeutic	1 (14.3)	3 (42.9)	3 (42.9)	7
In range	6 (20.7)	19 (65.5)	4 (13.8)	29
Supratherapeutic	8 (10.7)	26 (34.7)	41 (54.7)	75

Panel B. Dose increase (n = 30)

Pre-adjustment	Post <1.0	Post 1.0–5.0	Post >5.0	Total
Subtherapeutic	4 (33.3)	7 (58.3)	1 (8.3)	12
In range	4 (25.0)	12 (75.0)	0 (0.0)	16
Supratherapeutic	0 (0.0)	2 (100.0)	0 (0.0)	2

Values are n (row %). Concentrations in µg/mL. Subtherapeutic <1.0; in range 1.0–5.0; supratherapeutic >5.0.

Supplementary Table S11. Baseline characteristics by monitoring frequency (selection gradient)

Variable	1 TDM (n = 734)	2 TDM (n = 321)	≥3 TDM = cohort (n = 140)
Age, median (IQR), yr	57 (40–68)	55 (42–67)	62 (52–72)
Male, %	76	81	72
Infection department, %	42	50	33
Critical care, %	21	29	21
Liver disease, %[a]	5	9	9
First concentration <1.0 µg/mL (subtherapeutic), %	10	11	13
First concentration 1.0–5.0 µg/mL (in range), %	59	47	46
First concentration >5.0 µg/mL (supratherapeutic), %	31	42	41

Baseline characteristics of patients contributing one, two, or three or more valid TDM episodes; the ≥3 group in this table is the 140-patient genotyped analytic cohort used in the analyses, and 11 additional patients with ≥3 valid episodes lacked genotype data and were therefore not included in the analysed cohort. Baseline was taken as the first available TDM measurement. Patients with one or two episodes did not meet the repeated-monitoring criterion and are included only for selection comparison; CYP2C19 genotype was not available for these groups. [a]Liver disease in the one- and two-episode groups was parsed from free-text diagnoses and is likely undercounted, whereas the cohort value is a curated flag. Selection into repeated monitoring was associated mainly with out-of-range, and particularly supratherapeutic, first concentrations and with modestly older age, rather than with sex or departmental case-mix.
